# The Model of Motivational Dynamics in Sport: Resistance to Peer Influence, Behavioral Engagement and Disaffection, Dispositional Coping, and Resilience

**DOI:** 10.3389/fpsyg.2015.02010

**Published:** 2016-01-07

**Authors:** Adam R. Nicholls, David Morley, John L. Perry

**Affiliations:** ^1^Department of Sport, Health and Exercise Science, University of HullHull, UK; ^2^School of Education, Liverpool John Moores UniversityLiverpool, UK

**Keywords:** behavior, peers, mental toughness, motivation, motivational climate, sport

## Abstract

The Model of Motivational Dynamics (MMD; Skinner and Pitzer, 2012) infers that peers influence behavioral engagement levels, which in turn is linked to coping and resilience. Scholars, however, are yet to test the MMD among an athletic population. The purpose of this paper was to assess an *a priori* model that included key constructs from the MMD, such as resistance to peer influence, behavioral engagement and disaffection, coping, and resilience among athletes. Three hundred and fifty-one athletes (male *n* = 173, female *n* = 178; *M*_age_ = 16.15 years) completed a questionnaire that measured each construct. Our results provide support for the model. In particular, there were positive paths between resistance to peer influence and behavioral engagement, behavioral engagement and task-oriented coping, and task-oriented coping with resilience. There was also a positive path between resilience and resistance to peer influence, but a negative path from resistance to peer influence to behavioral disaffection. Due to the reported benefits of enhancing resistance to peer influence and behavioral engagement, researchers could devise sport specific interventions to maximize athletes’ scores in these constructs.

## Introduction

In order to promote life-long participation in sport, it is important that scholars create ways to maximize enjoyment for participants (Kirk, 2005). In order to do this, researchers need to identify the factors that influence enjoyment levels and manipulate those. Behavioral engagement is a psychological construct that is associated with enjoyment (Curran et al., 2013). Further, behavioral engagement is the key construct within Skinner and Pitzer’s (2012) Model of Motivational Dynamics (MMD), which infers that peers influence behavioral engagement levels, and that behavioral engagement is associated with both coping and resilience. In non-sport domains, Wentzel (1998) reported that behavioral engagement is influenced by peers. That is, peers can have a positive or negative influence on engagement levels. If peers are disruptive, they can negatively influence their friends, resulting in others becoming disaffected. Although scholars identified relationships between the aforementioned constructs, these were not explored among athletic populations and nor within the same model. Understanding more about the relationship between peer influence and engagement levels is important and may have important consequences for the organization of sports practice and competition, especially if peer influence is associated with engagement levels. Although, Skinner and Pitzer (2012) theorized an association between peers and behavioral engagement, along with both coping and resilience, scholars are yet to test the accuracy the MMD (Skinner and Pitzer, 2012) among athletes. The purpose of this paper was to assess the relevance of the MMD among an athletic population.

The MMD (Skinner and Pitzer, 2012) is grounded in self-determination theory and a model of positive motivational development. The central tenet of the MMD is that people will be engaged when their basic psychological needs are met. When a person’s basic psychological needs are not met, he or she will be behaviorally disaffected. Therefore, people need to feel connected to other people within the group and feel cared for, in control of their actions, and feel competent to be engaged (Ryan et al., 1996). A factor that influences whether a person will be behaviorally engaged or disaffected is peers, and thus the interactions a person has with his or friends and the pressure exerted by friends to influence behavior (Wentzel, 1998).

Indeed, peer pressure represents an important reason why people engage in delinquent or risky behavior (Simons-Morton et al., 2005). It is accepted that the main reason people engage in such delinquency is to impress their friends (Moffitt, 1993). One such mechanism that influences whether an individual will succumb to pressure to engage in delinquent behavior from peers is a person’s level of resistance to peer influence. This refers to the degree in which a person acts autonomously in interactions with their peers or friends (Steinberg and Monahan, 2007). Researchers from other domains of psychology suggested that resistance to peer influence is negatively associated with anti-social behavior (Monahan et al., 2009). Furthermore, the behavior of peers within a group may also influence behavioral engagement levels (Wentzel, 1998; Li et al., 2011). Indeed, Li et al. (2011) reported that negative peer relations detrimentally impacted upon behavioral engagement, and thus demonstrating the extent to which peers may influence behavioral engagement.

According to Curran et al. (2013), an individual is behaviorally engaged when he or she exhibits maximum effort and attention whilst performing an activity. Conversely, when an individual exerts little effort, he or she is behaviorally disaffected. Behavioral engagement and disaffection are important psychological constructs, because they predict learning, attendance, resilience, and achievement in school settings (Connell et al., 1994; Skinner et al., 1998). Furthermore, Shen et al. (2012) reported a positive association between behavioral engagement and relatedness to the teacher, within a physical education setting. That is, pupils who were more engaged felt more acceptance, belonging, and support from their teacher. It is important that psychologists understand more about the factors that influence engagement in order to maximize the likelihood of athletes having positive experiences whilst training or competing. One construct that might be affected by behavioral engagement is coping (Skinner and Pitzer, 2012).

Coping refers cognitive and behavioral efforts to manage internal or external demands (Lazarus and Folkman, 1984), and can be classified within task-, distraction-, and disengagement-oriented coping (Gaudreau and Blondin, 2004). During task-oriented coping the person attempts to change or master stressful situations, whereas distraction-oriented coping involves a person directing his or her attention onto unrelated aspects. Disengagement-oriented coping occurs when a person ceases efforts to attain his or her personal goals. Coping is a construct that appears to be related to many constructs such as performance (Doron and Gaudreau, 2014), goal attainment (Schellenberg et al., 2013), and choking under pressure (Balk et al., 2013). Skinner and Pitzer (2012) postulated that coping is related to behavioral engagement, suggesting that behavioral engagement acts as an energizing resource that enables people to cope more effectively with daily stressors. Disaffection, on the other hand, is associated with less effective coping.

Previous scholarly activity indicated that peers may impact upon on how an athlete copes. In particular, Nicholls et al. (2013) reported a negative path between peer-influence on behavior and distraction-oriented coping and a negative correlation between disengagement-oriented coping and peer influence on behavior. The negative path between peer influence on behavior and distraction-oriented coping could indicate that the athletes were too distracted by their peers to deploy task-oriented coping strategies. Although Nicholls et al. (2013) did not explore the relationship between coping and resistance to peer influence, their findings indicate that peers have an association with coping. Another construct that is thought to be related to coping is resilience (Fredrickson and Joiner, 2002).

Resilience represents a person’s ability to positively adapt to stressful situations and thereby function normally despite being exposed to stressful stimuli (Bonanno, 2004). Skinner and Pitzer (2012) suggested that repeated episodes of coping may influence a person’s mindset regarding perceptions of mastery and therefore resilience, given that mastery is a component of resilience (Yi-Frazier et al., 2009). Indeed, Yi-Frazier et al. (2009) found that maladaptive coping was associated with individuals who were not resilient. Understanding more about the relationship between coping and resilience will help scholars identify the strategies that are associated with resilience. This is important, given that coping is thought to enhance resilience (Skinner and Pitzer, 2012). Furthermore, resilience may also be related resistance to peer influence, as resilient individuals have been found to be less effected by peer victimizations (Overbeek et al., 2010).

**FIGURE 1:**
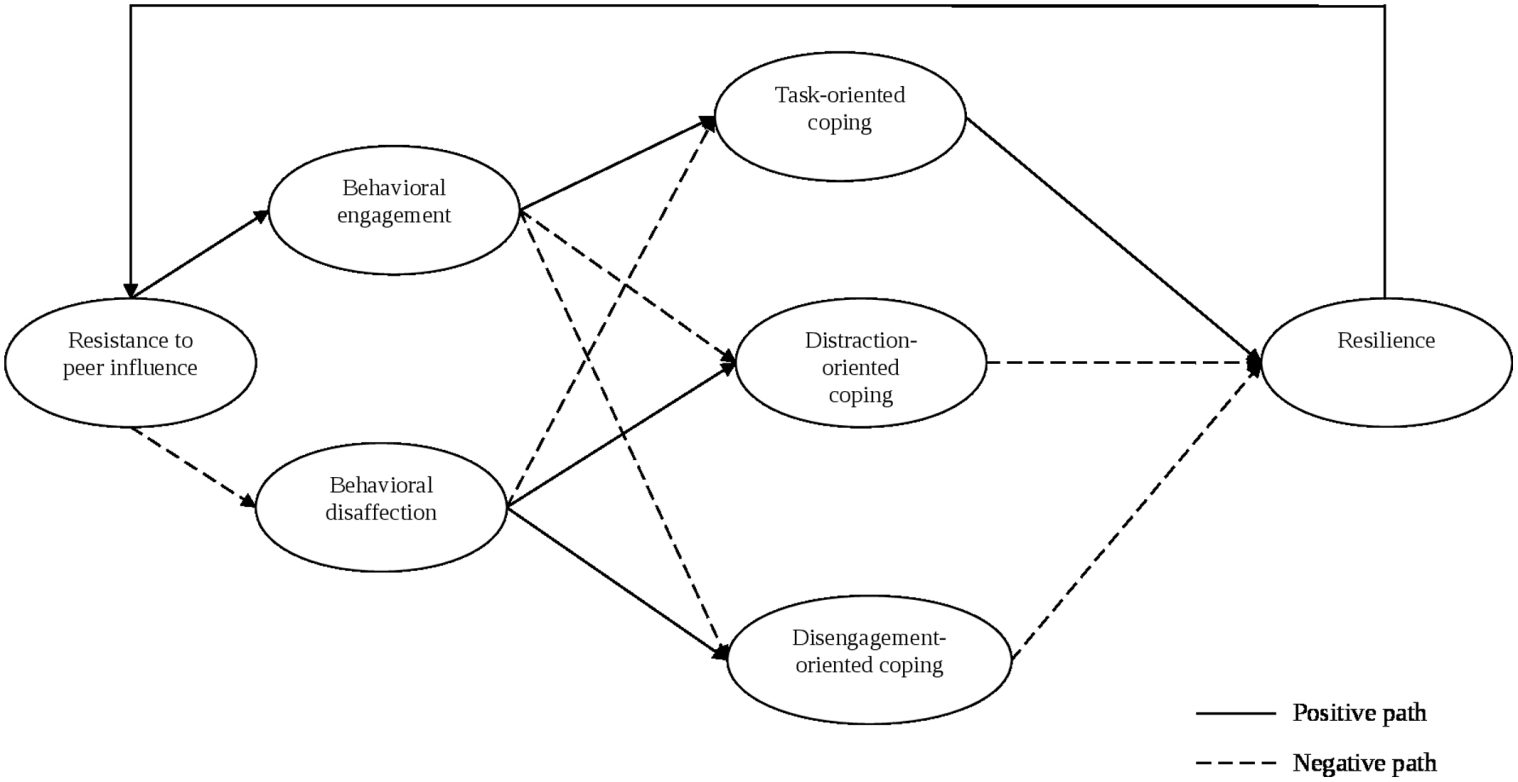
**Hypothesized model**.

The aim of this study was to examine the relevance of Skinner and Pitzer’s (2012) MMD among an athletic population. An illustrated version of our predicted paths is portrayed in **Figure 1**. A broken line infers a negative path, whereas an unbroken line infers a positive path. We predicted a positive path between resistance to peer influence and behavioral engagement, but a negative path between resistance to peer influence and behavioral disaffection. This is because researchers reported positive peer behaviors were associated with increased behavioral engagement, whereas negative peer behaviors were associated with behavioral disaffection (Wentzel, 1998). Indeed, Li et al. (2011) reported negative peer behaviors in school (i.e., bullying and being disruptive) caused behavioral disaffection in their longitudinal study. The ability to resist the temptation to engage in negative behaviors appears important in influencing whether an athlete is behavioral engaged or disaffected. We also hypothesized positive path between behavioral engagement and task-oriented coping, along with negative paths between behavioral engagement and both distraction- and disengagement-oriented coping. Positive paths between behavioral disaffection and both distraction- and disengagement-oriented coping in addition to a negative path between behavioral disaffection and task-oriented coping were also predicted. These paths are in accordance with Skinner and Pitzer’s (2012) MMD. These authors suggested that those who are behaviorally engaged are more likely to be focused, work hard, exert effort, search for strategies, and attempt to master challenging situations. Essentially, these behaviors are similar to those classified as task-oriented coping, but the antithesis of the behaviors and cognitions associated with distraction- or disengagement-oriented coping (Fredrickson and Joiner, 2002). Conversely, Skinner and Pitzer (2012) suggested that behavioral disaffection is associated with giving up and avoidance, which are akin to distraction- and disengagement-oriented coping strategies.

We also predicted a positive path between task-oriented coping and resilience, but negative paths between both distraction- and disengagement-oriented coping with resilience. We predicted these paths because maladaptive coping has been associated with individuals who are the least resilient (Yi-Frazier et al., 2009). Researchers in sport identified task-oriented coping as having adaptive outcomes and distraction-oriented coping strategies being associated with maladaptive outcomes (Nicholls et al., 2012; Schellenberg et al., 2013; Doron and Gaudreau, 2014). Finally, we predicted a positive path between resilience and resistance to peer influence, because resilient individuals are less likely to be influenced by their peers (Bonanno, 2004) and therefore not succumb to pressure from peers to behavior in a negative manner.

## Materials and Methods

### Participants

Three hundred and fifty-one athletes (male *n* = 173, female *n* = 178), aged between 11 and 31 years of age (*M*_age_ = 16.15, *s* = 4.28) participated in the study. Participants competed in team (*n* = 251) or individual sports (*n* = 100). The sample contained 333 Caucasian, four Asian, and 10 African-Caribbean, and four athletes from other ethnic origins. These athletes competed at international (*n* = 48), national (*n* = 38), county (*n* = 36), club (*n* = 88), and beginner (*n* = 140) levels.

### Measures

The Resistance to Peer Influence Scale (RPIS; Steinberg and Monahan, 2007) measured the extent to which the participants acted autonomously. The participants were instructed to read two conflicting statements for 10 different scenarios (e.g., “Some friends go along with their friends just to keep their friends happy” but “Other people refuse to go along with what their friends want to do, even though they know it will make their friends happy”). Participants were then asked to assess the level of their endorsement for ten different scenarios (e.g., *sort of true* or *really true)*. A higher score indicates that a person is less susceptible to being influenced by his or her peers. Steinberg and Monhan reported Cronbach’s alphas ranging from 0.70 to 0.76 among sub-samples of 3, 600 adolescents from lower income, detained, community, and serious offender groups. The ages of the participants ranged from 10 to 30 years-old.

The behavioral engagement and behavioral disaffection items from the Engagement versus Disaffection with Learning Scale (EDLS; Skinner et al., 2008) were used to assess behavioral engagement and disaffection. Participants completed a 10-items questionnaire that contained five behavioral engagement questions (e.g., “In training and competition, I try as hard as I can”) and five behavioral disaffection questions (e.g., “When I’m in training and competing, my mind wanders”). These questions were answered on a four-point Likert-type scale, which was anchored at 1 = *strongly disagree* and 4 = *strongly agree.* With a sample of 805 fourth to seven graders, Skinner et al. (2008) reported Cronbach’s alphas of 0.71 and 0.72 for the behavioral engagement subscale, along with 0.65 and 0.70 for the behavioral disaffection subscale.

The Dispositional Coping Inventory for Competitive Sport (DCICS; Hurst et al., 2011) assessed how the participants usually coped during training and competitions. The DCICS (Hurst et al., 2011) enables scholars to categorize coping within task-, distraction-, or disengagement-oriented coping dimensions. An example of a task-oriented question was “I give relentless effort,” whereas “I think about another hobby in order not to think about the competition” was a distraction-oriented question. “I let myself feel hopeless and discouraged” represents a disengagement-oriented coping question. Athletes were instructed to rate how they normally coped with the stress encountered in training or competition. Participants recorded their response on a five-point Likert-type scale, with 1 representing *“Does not correspond to what I do or think”* and five representing *“Corresponds very strongly to what I do or think.”* Hurst et al. (2011) reported Cronbach alpha coefficients for the 10 dispositional strategies ranging between 0.60 and 0.80, among a sample of 596 athletes. These athletes were aged between 18 and 23 years of age.

Finally, the Connor-Division Resilience Scale (CD-RISC; Campbell-Sills and Stein, 2007) measured resilience among the participants. The CD-RISC (Campbell-Sills and Stein, 2007) is a 10-items scale, which includes questions such as “I think of myself as a strong person when dealing with life’s challenges or difficulties” and “I am able to adapt when change occurs,” which are answered using a five-point Likert-type scale. These questions are anchored at 0 representing “*not true at all”* and 4 depicting “*true nearly all of the time.”* With a sample of 1,743 undergraduate students, Campbell-Sills and Stein (2007) reported a Cronbach alpha coefficient of 0.85.

### Procedure

A University Ethics Committee approved this study. After obtaining ethical approval, information letters were distributed to physical education teachers, coaches, sporting governing bodies, and sports clubs. This letter contained information relating to the study and the requirements of the participants. An information letter and assent form was sent to all participants who expressed a desire to take part in the research, and written assent was received by all participants before they could take part in the study. Informed consent forms were sent to the parents or guardians of the individuals who were aged 15 years and under. Written consent was obtained from parents for each participant, before they could take part. Participants received a standardized questionnaire pack and completed the questionnaires in the same order. All participants completed the RPIS (Steinberg and Monahan, 2007), 10 items from the EDLS (Skinner et al., 2008), the DCICS (Hurst et al., 2011), and then the CD-RISC (Campbell-Sills and Stein, 2007).

### Data Analysis

Data analysis included preliminary screening for outliers, normality, and checking of composite reliability. Correlation analyses involved examining coping strategies at the first (i.e., task-, distraction-, and disengagement-oriented) and second-order levels (e.g., mental imagery, effort expenditure, and distancing). We conducted structural equation modeling, using the two-step model building approach for the main analysis (Anderson and Gerbing, 1988). We tested the measurement model and then examined the hypothesized structural model, by adding the paths seen in **Figure 1**. All analyses were conducted using Mplus 7.1 (Muthén and Muthén, 2012), employing the robust maximum likelihood (MLR) estimator to guard against departure from multivariate normality. Measurement and structural models were assessed in accordance with Hu and Bentler (1999). As such, fit indices of CFI > 0.90, TLI > 0.90, SRMR < 0.08, RMSEA < 0.05 were deemed to represent an acceptable model fit, whereas CFI and TLI > 0.95 are indicative of an excellent model fit. We also note, however, the recommendations by Marsh et al. (2004), who correctly reminded researchers that the guidelines by Hu and Bentler (1999) should only be considered as general, rather than golden. To assess mediation, we examined direct and indirect effects. To interpret indirect effects, we used bootstrapping, as it does not hold assumptions of sampling distribution for indirect effects (Hayes, 2009). Further, bootstrapping generates standard errors and confidence intervals, enabling the researcher to examine invariance within a sample.

## Results

### Descriptive Statistics

Preliminary data screening identified no issues with missing data (<0.01%) or outliers. Univariate normality presented no issues for skewness (<2) or kurtosis (<2). In **Table 1** descriptive data is presented. Correlations between second-order coping strategies and resistance to peer influence, behavioral engagement, behavioral disaffection, and resilience are also presented. Most notably, all task-oriented coping strategies correlated positively with behavioral engagement (*r* = 0.24 to 0.44, *p* < 0.01). Low to moderate correlations were also found between all task-oriented coping strategies and resilience (*r* = 0.25 to 0.38, *p* < 0.01).

**Table 1:** Descriptive statistics, normality estimates, and second-order coping strategy correlations with resistance to peer influence, behavioral engagement, behavioral disaffection, and resilience.

	*M*	*SD*	Skewness	Kurtosis	ResPI	BE	BD	Res
Mental imagery	3.53	0.75	-0.27	-0.37	0.09	0.30ˆ**	-0.14ˆ**	0.34ˆ**
Effort expenditure	4.05	0.70	-0.54	-0.11	0.17ˆ**	0.44ˆ**	-0.10	0.31ˆ**
Thought control	3.58	0.65	-0.34	0.13	0.17ˆ**	0.24ˆ**	-0.11ˆ*	0.30ˆ**
Seeking support	3.06	0.82	-0.01	-0.11	0.09	0.30ˆ**	-0.18ˆ**	0.26ˆ**
Relaxation	2.95	0.89	0.11	-0.42	0.04	0.25ˆ**	-0.17ˆ**	0.25ˆ**
Logical analysis	3.40	0.77	-0.28	0.01	0.10	0.30ˆ**	-0.20ˆ**	0.38ˆ**
Distancing	1.90	0.86	0.80	-0.02	-0.04	-0.09	0.04	-0.13ˆ*
Mental distraction	2.28	0.80	0.21	-0.50	-0.16ˆ**	-0.13ˆ*	0.14ˆ*	-0.07
Venting emotions	2.69	0.84	0.30	-0.34	0.20ˆ**	0.02	-0.23ˆ**	-0.12ˆ*
Resignation	1.72	0.71	1.11	1.31	-0.18ˆ**	-0.23ˆ**	0.08	-0.23ˆ**

*ResPI, Resistance to peer influence; BE, behavioral engagement; BD, behavioral disaffection; Res, resilience*.

*^∗^*p* < 0.05; ^∗∗^*p* < 0.01*.

### Structural Equation Modeling

The complexity of the model relative to the sample size meant that a full latent analysis was not feasible. Bentler and Chou (1987) recommended at least five cases per estimated parameter to adequately test a hypothesized model. This appears to be the lower bound, with some authors suggesting a sample of at least 20:1 (Tanaka, 1987). Consequently, we adopted parceling for all latent variables in the hypothesized model (Bagozzi and Heatherton, 1994). Parceling involves reducing the number of path coefficients by collapsing items from a scale into multiple composites. In our model, each latent variable was indexed by two parcels. This resulted in a ratio between observations to free parameters of 5.57:1.

Parceling facilitates the identification of a structural equation model in which latent variables can be indexed by more than one indicator. This is a somewhat controversial technique, because it can be used to camouflage misspecifications in the measurement model (Marsh et al., 2013). To avoid such misspecification, we initially examined the factor structure of each measurement scale used in the study. Firstly, we subjected the RPIS (Steinberg and Monahan, 2007) to confirmatory factor analysis (CFA). This presented a good model fit: χ^2^(11) = 15.06, *p* = 0.130, CFI = 0.971, TLI = 0.940, SRMR = 0.029, RMSEA = 0.038 (90% CI = 0.000, 0.075). The CFA on the EDLS (Skinner et al., 2008) also yielded very good model fit: χ^2^(34) = 38.41, *p* = 0.202, CFI = 0.994, TLI = 0.992, SRMR = 0.031, RMSEA = 0.024 (90% CI = 0.000, 0.048). Thirdly, the DCICS (Hurst et al., 2011) presented a good absolute model fit: χ^2^(626) = 865.71, *p* < 0.001, SRMR = 0.063, RMSEA = 0.039 (90% CI = 0.034, 0.044), but with a marginal incremental fit: CFI = 0.896, TLI = 0.877. Finally, the factor structure of the CD-RISC (Campbell-Sills and Stein, 2007) also presented good model fit: χ^2^(35) = 62.12, *p* = 0.003, CFI = 0.951, TLI = 0.937, SRMR = 0.040, RMSEA = 0.047 (90% CI = 0.027, 0.066).

**FIGURE 2:**
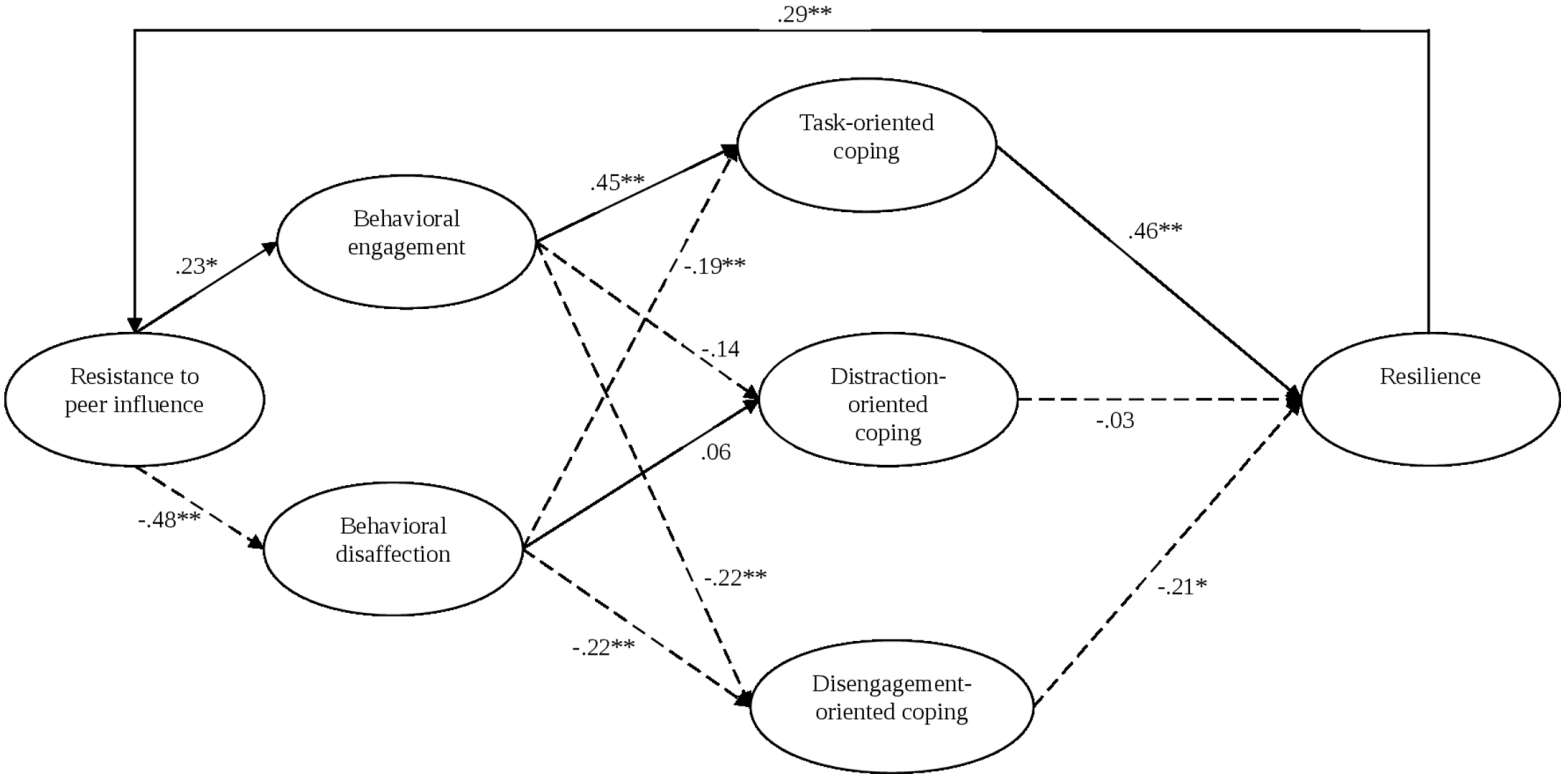
**Model of motivational dynamics in sport**.

All parcels were checked for normality, which presented no issues with univariate skewness or kurtosis. Examination of mean data suggested a potentially significant skill level effect (i.e., beginner, club, county, national, and international levels). To investigate this further, we conducted a one-way ANOVA with Tukey’s *post hoc* test and 2000 bootstrapped samples. Results confirmed a skill level effect for all latent variables except disengagement-oriented coping. Specifically, beginners were significantly [*F*(4,347) = 19.12, *p* < 0.01] less resistant to peer influence than all other groups. Beginners also reported significantly [*F*(4,347) = 13.10, *p* < 0.01] less behavioral engagement than all other skill levels, except county performers. International performers were more engaged than all other skill levels, except for the national athletes. In terms of behavioral disaffection, beginners were more disaffected than club, county, national, and international athletes [*F*(4,347) = 30.15, *p* < 0.01]. International performers reported significantly [*F*(4,347) = 13.12, *p* < 0.01] more task-oriented coping than beginner and club level participants. Beginners reported distraction-oriented corresponded more highly to how they coped than club, county, or international competitors [*F*(4,347) = 8.93, *p* < 0.01]. Finally, international athletes presented significantly [*F*(4,347) = 9.75, *p* < 0.01] greater resilience than all over skill level groups. As a result of the skill level differences, all SEM were controlled for skill. Age appeared to have little effect, as all variables correlated lowly with age (*r* < 0.25). The measurement model fit to the data at an acceptable level: χ^2^(63) = 98.82, *p* < 0.001, CFI = 0.975, TLI = 0.960, SRMR = 0.039, RMSEA = 0.047 (90% CI = 0.031, 0.062). Factor loadings and factor correlations are presented in **Table 2**. All standardized factor loadings were good (>0.60, *p* < 0.001). Moderately high correlations were evident for task-oriented coping with both behavioral engagement (*r* = 0.50, *p* < 0.01) and resilience (*r* = 0.50, *p* < 0.01). We then examined the structural model, which demonstrated a good model fit: χ^2^(71) = 135.40, *p* < 0.001, CFI = 0.964, TLI = 0.947, SRMR = 0.046, RMSEA = 0.051 (90% CI = 0.038, 0.064). Several of the structural paths were significant (see **Figure 2**). In particular, resistance to peer influence positively predicted behavioral engagement (β = 0.23, *p* < 0.01, 95% CI = 0.03, 0.43) and negatively predicted behavior disaffection (β = -0.48, *p* < 0.01, 95% CI = -0.64, -0.32). Task-oriented coping was significantly predicted by behavioral engagement (β = 0.45, *p* < 0.01, 95% CI = 0.30, 0.61) and negatively predicted by behavioral disaffection (β = -0.19, *p* < 0.01, 95% CI = -0.27, -0.01). Resilience was positively predicted by task-oriented coping (β = 0.46, *p* < 0.01, 95% CI = 0.26, 0.67) but negatively predicted by disengagement-oriented coping (β = -0.21, *p* < 0.05, 95% CI = -0.47, 0.05). Finally, resilience completed the cyclical model by positively predicting resistance to peer influence (β = 0.29, *p* < 0.01, 95% CI = 0.00, 0.43).

**Table 2:** Factor loadings, composite reliability, and factor correlations.

Variable	P1 FL	P2 FL	1	2	3	4	5	6	7
(1) Resistance to peer influence	0.71	0.61	(0.65)						
(2) Behavioral engagement	0.84	0.83	0.20	(0.75)					
(3) Behavioral disaffection	0.77	0.98	0.28ˆ*	-0.28ˆ**	(0.78)				
(4) Task-oriented coping	0.90	0.91	0.12	0.50ˆ**	-0.23ˆ**	(0.84)			
(5) Distraction-oriented coping	0.68	0.82	-0.18	-0.20ˆ**	0.20ˆ**	-0.06	(0.62)		
(6) Disengagement-oriented coping	0.70	0.83	-0.26ˆ*	-0.17ˆ*	-0.09	-0.20ˆ**	0.47ˆ**	(0.66)	
(7) Resilience	0.85	0.77	0.31ˆ*	0.39ˆ**	-0.03	0.50ˆ**	-0.18ˆ**	-0.28ˆ**	(0.70)

*P1 and P2 refer to composite parcels. FL, factor loading. Composite reliabilities shown in parentheses. Factor correlations are taken from measurement model*.

*^∗^*p* < 0.05; ^∗∗^*p* < 0.01*.

To examine mediation effects within our model, we calculated direct and indirect effects using the maximum likelihood estimator and a bootstrap of 5,000 replications for confidence intervals. The absence of zero in the confidence intervals indicates a significant indirect effect. The only indirect effect that met this criterion was the path between behavioral engagement and resilience, mediated by task-oriented coping, which was a positive indirect effect (γ = 0.17, *p* < 0.01, 95% CI = 0.05, 0.29). This mediation was partial, because the direct path was also significant (β = 0.24, *p* < 0.01, 95% CI = 0.05, 0.46).

To explore potential gender impact on the model, a series of nested models were tested for measurement and structural invariance. This is the extent to which substantive invariance remains across subsamples on a series of increasingly constrained models. First, configural invariance was assessed by replicating the parceled measurement model across groups. Second, factors were constrained to test metric invariance. Third, we examined scalar invariance by constraining factors and item intercepts. Fourthly, structural invariance was tested by constraining structural paths while maintaining scalar invariance. As a general rule, ΔCFI ≤ 0.01 supports invariance (Cheung and Rensvold, 2002). Results are displayed in **Table 3**, which supports invariance.

**Table 3:** Measurement and structural invariance for gender.

Model	χ^2^	*df*	Δ χ^2^	Δ*df*	CFI	TLI	SRMR	RMSEA (90% CI)
**Gender**								
Configural invariance	256.79	128	–	–	0.929	0.899	0.070	0.076 (0.062, 0.089)
Metric invariance	262.21	135	5.42	7	0.930	0.905	0.079	0.073 (0.060, 0.087)
Scalar invariance	270.88	142	8.67	7	0.929	0.909	0.078	0.072 (0.059, 0.085)
Structural invariance	292.81	149	21.93	7	0.920	0.903	0.081	0.074 (0.062, 0.087)

## Discussion

In this paper we assessed the relevance of the MMD (Skinner and Pitzer, 2012) within a sporting context. As such, we tested a model that included resistance to peer influence, behavioral engagement and disaffection, dispositional coping, and resilience among a sample of athletes. Overall, many of our hypotheses were supported and there was a strong model fit, thus illustrating the relevance of the MMD in sport settings. In particular, there were positive paths between resistance to peer influence and behavioral engagement, behavioral engagement and task-oriented coping, task-oriented coping and resilience, and resilience with resistance to peer influence. There was also a negative path between resistance to peer influence and behavioral disaffection.

In accordance with the MMD (Skinner and Pitzer, 2012), we found a positive path between resistance to peer influence and behavioral engagement, but a negative path between resistance to peer influence and behavioral disaffection. In other sporting contexts, scholars revealed the importance of athletes being able to resist social influences from peers as a key factor that determines whether an athlete intends to take performance enhancing drugs (i.e., Lucidi et al., 2008). In addition to impacting upon doping, findings from the present study also indicate that resistance peer influence may impact upon whether an athlete is behaviorally engaged or disaffected, although experimental research is required to infer causality between these constructs. Nevertheless, the influence of peers appears to be strong and can affect an athlete either positively or negatively, so coaches and sport psychologists could be aware of peer influence. Enhancing an athlete’s resistance to negative peer behaviors may be important in promoting positive behaviors such as enhancing behavioral engagement or reducing intentions to dope, particularly among lower skilled athletes who are less able to resist negative peer influence.

The positive path between behavioral engagement and task-oriented coping provides support for Skinner and Pitzer’s (2012), who outlined the behaviors and cognitive orientations of those who are behaviorally engaged in their MMD. There are potentially important implications of this finding. Fostering behavioral engagement in the sporting environment may result in athletes employing task-oriented coping strategies, which linked to more adaptive performance outcomes (Gaudreau et al., 2010; Doron and Gaudreau, 2014), goal attainment Schellenberg et al. (2013), and coping effectiveness (Nicholls et al., 2010). However, interventions specifically designed to maximize behavioral engagement among athletes are non-existent.

Based on our findings, the MMD (Skinner and Pitzer, 2012) may be a useful theoretical framework to enhance behavioral engagement among athletes. An MMD guided intervention to enhance behavioral engagement would be concerned with enhancing autonomy, competence, and relatedness among athletes and thus meeting an athlete’s psychological needs. In addition to enhancing behavioral engagement, an indirect benefit of such an intervention may be enhanced mental toughness. Mahoney et al. (2014) recently found a positive association between the extent to which a person’s basic psychological needs are met and mental toughness (Mahoney et al., 2014). Experimental research and theory guided interventions, based on the MMD (Skinner and Pitzer, 2012), are warranted to establish causality and the efficacy of such an intervention in sport. This will enable scholars to understand whether behavioral engagement can be enhanced and whether this results in enhanced behavioral engagement and other related constructs such as more frequent positive experiences (Skinner and Pitzer, 2012) and enhanced mental toughness (Mahoney et al., 2014).

Alternatively, scholars could also examine the effects of shaping the motivational climate to enhance behavioral engagement. It is plausible that peer influence may shaped by the motivational climate. In task-oriented climates, individuals are praised for effort and improvement, so athletes are less likely to be influenced by peers in comparison with ego-oriented climates, because success is not judged in relation to peers (Nicholls, 1989). In support of this idea, Skinner and Pitzer (2012) suggested that behavioral engagement could be enhanced via promoting mastery climates, where hard work and improvement are encouraged. Scholars such as Hogue et al. (2013) demonstrated that mastery climates can be developed in a sport setting, so it would therefore be interesting to see if such an intervention could enhance behavioral engagement.

The hypothesized significant and positive path between task-oriented coping and resilience occurred, but the predicted paths between resilience and both distraction- and disengagement-oriented coping were insignificant. The positive path between task-oriented coping and resilience is in agreement with Yi-Frazier et al. (2009), in that adaptive coping strategies were associated with resilience. Enhancing resilience may have a positive impact on performance following any adversity that athletes encounter, because Seligman et al. (1990) found that swimmers who coaches rated as being more resilient performed better after adversity. Increasing resilience through coping interventions may also positively impact resistance to peer influence too. The results from this study, which were in support of our hypothesis, yielded a positive path between resilience and resistance to peer influence. Increasing resistance to peer influence could potentially have desirable effects, such as minimizing the negative effects that peers have on development (Altermatt and Pomerantz, 2003) and achievement (Chen et al., 2003). Further, Nicholls et al. (2013) revealed a negative path between peer influence on behavior and distraction-oriented coping. This form of coping has been associated negatively with goal attainment (Schellenberg et al., 2013). Minimizing the effects that peers may have on other individuals may decrease the use of distraction-oriented coping, which could have a positive impact on goal attainment.

A hypothesis not supported was the path between behavioral disaffection and disengagement-oriented coping. We hypothesized a positive path between these constructs. There was, however, a significant negative path between behavioral disaffection and disengagement-oriented coping. It could be that the most disaffected athletes did not report using disengagement-oriented coping strategies, because they did not have any goals to disengage from in the first place or that they were resigned to not achieving their goals, so have already disengaged before competition or training starts. In support of this idea, Skinner and Pitzer (2012) suggested that behaviorally disaffected individuals can be aimless and resigned, which infers these people do not have any goals or have accepted defeat in attempts to achieve their goals. Although psychologists have an important role in maximizing behavioral engagement, they are also required to minimize behavioral disaffection, given that this is construct is negatively associated with basic needs satisfaction (Curran et al., 2013) and a range of unpleasant emotions such as sadness, anxiety, shame, and boredom (Skinner and Pitzer, 2012). Indeed, Skinner and Pitzer provided some ideas regarding how behavioral disaffection can be reduced or eliminated, such as, tracking people and monitoring disaffection levels. If disaffection levels are high among certain individuals, high scores should be seen as cues to increase warmth, involvement, autonomy support, and structure toward particular individuals.

A possible limitation of this research is that three of the four questionnaires used in this study have not been validated among athletic samples. This might be a limitation, as Nicholls et al. (2014) found problems with reliability when using non sport-specific measures with an athletic sample. However, the constructs measured in this study have important implications for athlete well-being, so we thought it was acceptable to use these questionnaires. We also used a cross-sectional design, which means we cannot infer causality.

## Conclusion

We found support for Skinner and Pitzer’s (2012) MMD within a sport setting. That is, resistance to peer influence was positively associated with behavioral engagement, whereas resistance to peer influence was negatively associated with behavioral disaffection. Behavioral engagement was positively associated with task-oriented coping, and task-oriented coping was associated with resilience. Further, resilience was positively associated with resistance to peer influence. Given the reported benefits of enhancing resistance to peer influence and behavioral engagement, scholars could conduct experimental studies to examine causality between these constructs, track fluctuations in these constructs over time, and devise sport specific interventions to maximize athletes’ scores in these constructs.

## Conflict of Interest Statement

The authors declare that the research was conducted in the absence of any commercial or financial relationships that could be construed as a potential conflict of interest.
